# Functional tooth number in the posterior region associated serum ucOC levels

**DOI:** 10.1186/s40729-022-00450-2

**Published:** 2022-11-01

**Authors:** Masahiko Nakamura, Yusuke Kondo, Tetsuji Nakamoto, Fumiko Nakagawa Aonuma, Tomotaka Nodai, Takashi Munemasa, Taro Mukaibo, Chihiro Masaki, Ryuji Hosokawa

**Affiliations:** 1 Division of Oral Reconstruction and Rehabilitation, Kyushu Dental University, 2-6-1 Manazuru, Kokurakita-Ku, Kitakyushu, Fukuoka 803-8580 Japan; 2 Department of Maxillofacial Implant, Asahi University School of Dentistry, Mizuho, Japan

**Keywords:** Osteocalcin, Undercarboxylated osteocalcin (ucOC), Vitamin K, Functional tooth number

## Abstract

**Purpose:**

The purpose of this study was to investigate serum undercarboxylated osteocalcin (ucOC) levels in partially edentulous patients scheduled to receive implant treatment and determine the association between ucOC levels, vegetable intake, vitamin K, dietary fiber intake, and functional tooth number in the posterior region (p-FTN).

**Methods:**

A total of 46 patients (20 male and 26 female, 61.9 ± 12.7 years old) were included. The association among serum ucOC levels, vegetable intake, vitamin K and dietary fiber intake was assessed using Spearman’s rank correlation coefficient and binary logistic regression analysis.

**Results:**

In total, 35% of patients (16/46 subjects) showed an abnormally high ucOC level (≧ 4.5 ng/mL). p-FTN showed a weak positive correlation with vegetable intake, vitamin K and dietary fiber intake (*r* = 0.28, 0.21, and 0.14, respectively) and a significant negative correlation with ucOC levels (*r* = − 0.51). Multivariate analysis demonstrated that p-FTN as well as vitamin K intake showed a significant negative association with serum ucOC levels.

**Conclusions:**

More than one-third of patients showed abnormally high ucOC levels. p-FTN showed a negative association with serum ucOC levels, which indicated the possibility that oral status affected bone quality.

## Background

Dental implant treatment indicates a high survival rate, and significantly improves oral health-related quality-of-life [1]. Direct structural and functional connection between the living bone and implant surface is fundamental to implant treatment [2]. To assess bone condition, CT scan-based classification is commonly used [3]. On the other hand, the National Institutes of Health proposed that bone strength primarily reflects the integration of bone density and bone quality. Additionally, architecture, turnover, damage accumulation, and mineralization were listed as factors that determine bone quality [4]. However, a previous study reported the application of bone turnover markers (BTMs) for implant treatment [5], but sufficient evidence had not been established and further studies were required.

Osteocalcin (OC) is one of the BTMs produced by osteoblasts and the most abundant non-collagenous protein in bone [6]. OC undergoes g-carboxylation by vitamin K, and glutamic acid (Glu) residues are converted into γ-carboxyglutamic acid (Gla) [7]. Carboxylated osteocalcin binds calcium directly. However, undercarboxylated osteocalcin (ucOC) lacks the ability to bind to the mineral hydroxyapatite, which is released into the circulation [8]. Consequently, serum ucOC levels are negatively correlated with vitamin K intake, and are used clinically as an indicator of vitamin K status [9]. Vitamin K consists of vitamins K1, 2, 3, 4, and 5. Both vitamin K1 (phylloquinone) and vitamin K2 (menaquinones) occur in nature. Green leafy vegetables and seaweeds are rich in vitamin K1, whereas fermented foods are rich in vitamin K2, and bacteria in the gut also synthesize vitamin K2 [10, 11]. Higher ucOC levels are related to lower bone mineral density (BMD) as well as a higher risk of hip fracture [12, 13]. Furthermore, Tanaka et al. found an association between higher ucOC and poor bone strength using quantitative ultrasound in women [14].

The serum ucOC level is affected by several factors. For example, warfarin sodium increases the serum ucOC level through the inhibition of vitamin K functions [15]. In contrast, other medications, such as corticosteroids and bone resorption inhibitors, can decrease the serum ucOC level [16]. Furthermore, it was reported that gastrectomy and Crohn's disease increased serum ucOC levels, which suggested that digestive hypofunction resulted in increased undercarboxylated osteocalcin through vitamin K hypoabsorption and alteration of intestinal flora [17, 18]. Oral function plays a critical role in digestion, and it affects food variety intake and intestinal flora, therefore oral function may also be associated with serum ucOC levels; however, this has yet to be proven.

In this study, we aim to determine insights into ucOC levels in partially edentulous patients scheduled to receive implant treatment, and the association between serum ucOC levels, vitamin K intake and functional tooth number in the posterior region (p-FTN).

## Methods

### Study population

This study included 46 patients (20 male and 26 female, 61.9 ± 12.7 years old) drawn from the Kyushu Dental University Hospital in Japan. The inclusion criteria included partially edentulous patients scheduled to receive implant treatment in the posterior region and had consented to participate in this study. The exclusion criteria included: (1) patients prescribed warfarin sodium or corticosteroids; (2) patients prescribed osteoporosis treatment; (3) patients with gastric or intestinal resection; (4) patients who wore a removable partial denture; (5) patients with anterior tooth loss and without a prosthesis in the anterior region; (6) patients with one or more teeth with “hyper mobility” (class 3 mobility according to Miller’s classification) [19]; and (7) participants who had not given consent.

### Outcomes

p-FTN included functional natural tooth, pontic and dental implant crown in the posterior region, and excluded remaining root and wisdom tooth. Vegetable intake, vitamin K, and dietary fiber intake were assessed using a brief self-administered diet history questionnaire (BDHQ). The BDHQ is a questionnaire that consisted of 75 questions (55 questions that related to food consumption and 17 to cooking and dietary behaviors); it assessed dietary habits over the past month [20]. Nutrient intake was calculated from the results of the BDHQ questionnaire using an ad hoc computer algorithm [21]. In order to compensate for declaration error, results were energy-adjusted using estimated energy requirement; energy-adjusted intake = intake /energy intake × estimated energy requirement. Serum ucOC levels were measured by electrochemiluminescence immunoassay.

### Statistical analyses

Spearman’s rank correlation coefficient and binary logistic regression analysis were performed. In binary logistic regression analysis, the binary outcomes of abnormally high ucOC (≧ 4.5 ng/mL) = 1 and normal ucOC (< 4.5 ng/mL) = 0 were employed as the dependent variable. Statistical analyses were computed using BellCurve for Excel (Social Survey Research Information Co., Ltd., Tokyo, Japan); α = 0.05 was considered significant.

## Results

### Subject characteristics and outcomes

The characteristics of subjects and outcomes are shown in Table 1. The median p-FTN, vegetable intake, vitamin K, dietary fiber intake, and serum ucOC levels were 13, 255.1 g/day, 356.0 μg/day, 13.9 g/day, and 3.94 ng/mL, respectively (Table 1). The percentage of subjects who did not reach the recommended volume was 82.3% (38/46 subjects), 26.1% (12/46 subjects), and 91.3% (42/46 subjects) for vegetable intake (recommended volume in Japan: 350 g/day), vitamin K (recommended volume for osteoporosis prevention in Japan: 250 μg/day) and dietary fiber (recommended volume in Japan: male 21 g/day, female: 18 g/day), respectively. The ucOC levels in 35% of subjects (16/46 subjects) showed an abnormally high value (≧ 4.5 ng/mL) [22]).

**Table 1:** Characteristics of subjects and outcomes

Age (years old)	63 (54.25–70.75)
Sex	Male 20 female 26
p-FTN	13 (12–14)
Vegetable intake (g/day)	255.1 (181.9–333.1)
Vitamin K intake (μg/day)	356.0 (260.9–416.8)
Dietary fiber intake (g/day)	13.9 (11.4–16.4)
ucOC (ng/mL)	3.94 (2.89–5.38)

Data show median (first quartile, third quartile) or the number of people

### Correlation between outcomes

**Fig. 1:**
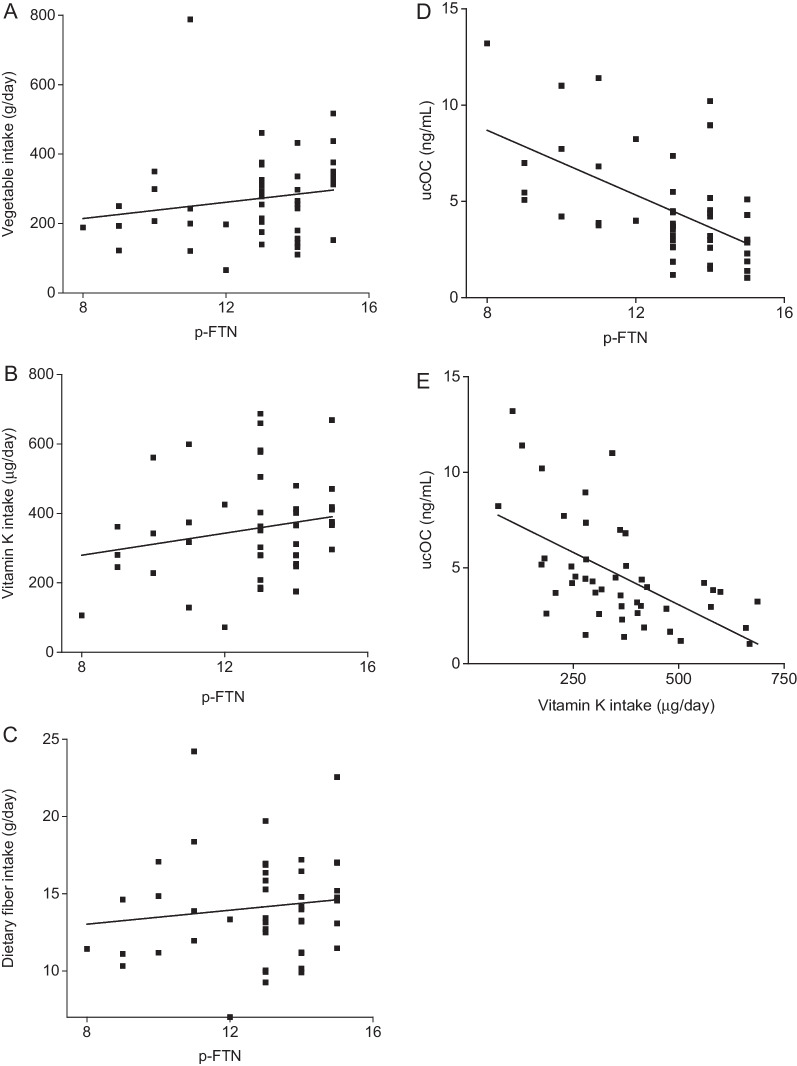
Correlation between outcomes. **A** Correlation between p-FTN and vegetable intake (*r* = 0.28, *p* = 0.064). **B** Correlation between p-FTN and vitamin K (*r* = 0.21, *p* = 0.17). **C** Correlation between p-FTN and dietary fiber intake (*r* = 0.14, *p* = 0.36). **D** Correlation between p-FTN and ucOC level (*r* = − 0.51, *p* < 0.001). **E** Correlation between vitamin K intake and ucOC level (*r* = − 0.60, *p* < 0.001)

The correlation diagram between p-FTN, vegetable intake, vitamin K intake, dietary fiber intake, and ucOC levels, and results from the Spearman’s rank correlation coefficient are shown in Fig. 1 and Table 2. p-FTN showed a weak positive correlation with vegetable intake, vitamin K and dietary fiber intake (*r* = 0.28, 0.21, and 0.14, respectively). p-FTN, vegetable intake, vitamin K and dietary fiber intake showed a significant negative correlation with ucOC levels (*r* = − 0.51, − 0.35, and − 0.34, respectively). Vitamin K and dietary fiber intake were strongly associated with vegetable intake (*r* = 0.69 and 0.75, respectively).

**Table 2:** Correlations between outcomes (Spearman’s rank correlation coefficient)

		p-FTN	Vegetable intake	Vitamin K intake	Dietary fiber intake	ucOC
p-FTN	Correlation coefficient	–	0.28	0.21	0.14	− 0.51
	*p*-value		0.064	0.17	0.36	< 0.001
Vegetable intake	Correlation coefficient		–	0.69	0.75	− 0.35
	*p*-value			< 0.001	< 0.001	0.019
Vitamin K intake	Correlation coefficient			–	0.72	− 0.60
	*p*-value				< 0.001	< 0.001
Dietary fiber intake	Correlation coefficient				–	− 0.34
	*p*-value					0.020
ucOC	Correlation coefficient					–
	*p*-value					

### Multivariate analysis of serum ucOC

Binary logistic regression analysis was used to identify the relationship between ucOC levels and other outcomes and age and sex as confounders. The results indicated that both p-FTN [odds ratio = 0.60, 95% confidence interval (CI) = 0.38–0.95] and vitamin K intake (odds ratio = 0.18, 95%CI = 0.035–0.95) showed significant negative association with ucOC levels (Table 3).

**Table 3:** Binary logistic regression analysis with serum ucOC level as the dependent variable

Covariate	Odds ratio	*p*-value	95% confidence interval
Age	0.97	0.35	0.91–1.04
Sex			
Male: 1 Female: 0	0.32	0.18	0.062–1.70
p-FTN	0.60	0.030	0.38–0.95
Vitamin K intake			
≧ 250 μg: 1 < 250 μg: 0	0.18	0.043	0.035–0.95

Abnormally high ucOC (≧ 4.5 ng/mL): 1, normal ucOC (< 4.5 ng/mL): 0

## Discussion

The use of BTMs indicated bone formation and resorption, which has an important role in the management of osteoporosis [23]. During implant treatment, systematic diseases that impair bone turnover, such as diabetes, exacerbate the peri-implant tissue conditions [24]. A recent study that investigated BTMs in subjects who were scheduled to receive implant treatment demonstrated that almost 50% of subjects showed abnormal BTMs, and bone-specific alkaline phosphatase (BAP) and cross-linked N-telopeptide of type I collagen (NTX) were frequently abnormally high [5]. Our results show that more than one-third of partial edentulous subjects show abnormally high ucOC, which suggests that clinicians must improve awareness of ucOC levels in advance of implant treatment. It was reported that nutritional therapy with vitamin K1 effectively decreases ucOC [25]. Vitamin K2 intake from fermented food represented by Natto could also decrease ucOC [26]. Furthermore, vitamin K2 enabled bone microstructural and mechanical properties in the ovariectomized model rat [27]. Considering the above, preimplant-surgery dietary counseling should be considered.

Although a lot of studies have investigated the association between ucOC, vitamin K, bone quality, and related factors, this study is the first report to suggest the possibility that oral status affects serum ucOC levels. Tooth loss affects food intake and decreases vegetable intake [28, 29]. Vegetable intake, especially green leafy vegetables, are rich in vitamin K1, decreased p-FTN might induce high ucOC in this study. Decreased vegetable intake results in both reduced vitamin K intake and dietary fiber intake [28–30]. It is known that dietary fiber consumed by gut bacteria affects the composition and diversity of gut microbiota as well as microbial metabolic activities, including the production of fermentative end products [31]. Although the direct association between dietary fiber consumption and vitamin K production has not been previously described, the present results suggest that increased dietary fiber intake potentially decreases ucOC through enhanced vitamin K production in the gut. Future studies that include microbiota analysis are required.

It was reported that gastrectomized patients showed lower BMD and higher fracture risk than those without [32]. Vitamin D deficiency and secondary hyperparathyroidism have been proposed as common mechanisms for BMD impairment in gastrectomized patients [33]. Alternately, some studies proposed an association between ucOC and vitamin K dissolution and absorption: Ivaska et al. and Iki et al. found abnormal high ucOC in patients with a history of gastrectomy, and it was speculated that an elided or a shortened passage through the duodenum resulted in reduced absorption of vitamin K [12, 34]. The dissolution into dietary lipid is required to absorb lipid-soluble vitamins, including vitamin K [35], and oral function, including crushing and mixing food, plays an important role in lipid-soluble vitamin dissolution. Therefore, decreased p-FTN could induce high ucOC via impaired vitamin K dissolution and absorption. Several previous studies had demonstrated a negative association between tooth number and osteoporosis status [36–38]; however, the underlying mechanism was unknown. Results of the present study demonstrate the possibility that tooth loss may induce the progression of osteoporosis due to the increase in ucOC levels.

There are several limitations to this study. The absence of serum vitamin K level is a limitation of this study. Serum ucOC measurement is a well-established method used in clinical examination and research as a biochemical marker of vitamin K insufficiency. Hence, we measured the serum ucOC level in this study. In contrast, serum vitamin K fraction analysis can evaluate vitamin K1 and K2, including the subtypes. Further study should include vitamin K fraction analysis to provide more details on how p-FTN associates with vitamin K. Our results demonstrated an association between p-FTN and vitamin K insufficiency. However, as this was a cross-sectional study, a causal relationship cannot be proven. Furthermore, it remains unknown if prosthodontic interventions contribute to a decrease in the serum ucOC level. Future longitudinal and intervention studies are warranted.

## Conclusions

In this study, we demonstrate that p-FTN, as well as vitamin K levels, exhibit a negative association with serum ucOC levels. It is probable that oral status impacts bone quality through ucOC levels.

## Data Availability

The data that support the findings of this study are available from the corresponding author upon reasonable request.
